# Exploring Current Practices, Needs, and Barriers for Expanding Distributed Medical Education and Scholarship in Psychiatry: Protocol for an Environmental Scan Using a Formal Information Search Approach and Explanatory Design

**DOI:** 10.2196/46835

**Published:** 2023-11-27

**Authors:** Lara Hazelton, Raquel da Luz Dias, Mandy Esliger, Philip Tibbo, Nachiketa Sinha, Anthony Njoku, Satyendra Satyanarayana, Sanjay Siddhartha, Peggy Alexiadis-Brown, Faisal Rahman, Hugh Maguire, Gerald Gray, Mark Bosma, Deborah Parker, Owen Connolly, Adewale Raji, Alexandra Manning, Alexa Bagnell, Vincent Israel Opoku Agyapong

**Affiliations:** 1 Department of Psychiatry Faculty of Medicine Dalhousie University Halifax, NS Canada; 2 Department of Psychiatry Faculty of Medicine Dalhousie University Saint John, NB Canada; 3 Mental Health and Addictions Program Nova Scotia Health Antigonish, NS Canada; 4 Mental Health and Addictions Program Nova Scotia Health Truro, NS Canada; 5 Mental Health and Addictions Program Nova Scotia Health Kentville, NS Canada; 6 Child and Adolescent Psychiatry IWK Health Halifax, NS Canada

**Keywords:** distributed learning sites, medical education, psychiatry, environmental scan, needs assessment, strategic plan, distributed medical education, rural area, physician, mixed methods approach, education program

## Abstract

**Background:**

Distributed medical education (DME) offers manifold benefits, such as increased training capacity, enhanced clinical learning, and enhanced rural physician recruitment. Engaged faculty are pivotal to DME's success, necessitating efforts from the academic department to promote integration into scholarly and research activities. Environmental scanning has been used to gather, analyze, and apply information for strategic planning purposes. It helps organizations identify current practices, assess needs and barriers, and respond to emerging risks and opportunities. There are process models and conceptual frameworks developed for environmental scanning in the business and educational sectors. However, the literature lacks methodological direction on how to go about designing and implementing this strategy to guide research and practice in DME, especially in the psychiatry field.

**Objective:**

This paper presents a protocol for an environmental scanning that aims to understand current practices and identify needs and barriers that must be addressed to facilitate the integration of psychiatrists from the Dalhousie University Faculty of Medicine’s distributed education sites in Nova Scotia and New Brunswick into the Department of Psychiatry, contributing for the expansion of DME in both provinces and informing strategic planning and decision-making within the organization.

**Methods:**

This protocol adopts an innovative approach combining a formal information search and an explanatory design that includes quantitative and qualitative data. About 120 psychiatrists from 8 administrative health zones of both provinces will be invited to complete an anonymous web-based survey with questions about demographics, participants' experience and interest in undergraduate, postgraduate, and continuing medical education, research and scholarly activities, quality improvement, and knowledge translation. Focus group sessions will be conducted with a purposive sample of psychiatrists to collect qualitative data on their perspectives on the expansion of DME.

**Results:**

Results are expected within 6 months of data collection and will inform policy options for expanding Dalhousie University’s psychiatry residency and fellowship programs using the infrastructure and human resources at distributed learning sites, leveraging opportunities regionally, especially in rural areas.

**Conclusions:**

This paper proposes a comprehensive environmental scan procedure adapted from existing approaches. It does this by collecting important characteristics that affect psychiatrists' desire to be involved with research and scholarly activities, which is crucial for the DME expansion. Furthermore, its concordance with the literature facilitates interpretation and comparison. The protocol's new method also fills DME information gaps, allowing one to identify insights and patterns that may shape psychiatric education. This environmental scan's results will answer essential questions about how training programs could involve therapists outside the academic core and make the most of training experiences in semiurban and rural areas. This could help other psychiatry and medical units outside tertiary care establish residency and fellowship programs.

**International Registered Report Identifier (IRRID):**

DERR1-10.2196/46835

## Introduction

### Distributed Medical Education

The need to recruit physicians into rural areas and regional centers in Canada is well-recognized. According to a report by Pong et al [1], 9.4% of all Canadian physicians were practicing in rural areas compared with 21.1% of Canadians living in rural and small towns. One approach that has been used to successfully recruit and retain trainees in underserved communities is distributed medical education (DME) [2-6]. DME was first used in postgraduate medical education (PGME) in the 1970s to recruit and retain trainees in targeted rural communities. In the intervening years, it has been widely incorporated into undergraduate medical education (UGME) and PGME [7-9]. Studies have shown that longer rural postgraduate training is strongly associated with rural practice among general practitioners [10-12]. Preliminary studies have also suggested that increased exposure to rural practice in psychiatry residency training programs may increase the number of trainees choosing to practice in these communities after graduating [13,14].

In addition to the potential benefit of recruiting physicians, rural training offers trainees unique clinical experiences and provides first-hand knowledge of the health care systems that exist in rural, small, and underserved communities located outside of the academic centers in regional or “satellite” sites [13,15]. There is also the possibility that exposure to smaller communities and community-based practice may encourage generalism [16]. The Royal College of Physicians and Surgeons of Canada defines generalism as “a commitment to a breadth of practice within one’s discipline and responsiveness to patient needs” [17] and suggests that specialty training should include rotations in community-based centers in order to expose trainees to generalism and its importance. Adopting a generalist approach enables physicians to manage uncertainty, approach undifferentiated problems, and incorporate a broad understanding of disease prevalence and psychosocial context into the presenting health care concerns and management of patients [18].

While there is strong evidence regarding the value of distributed education, it is important to recognize that the successful implementation of DME involves more than simply sending trainees to distributed sites. In advance of any expansion of training, it is important to develop a comprehensive understanding of the opportunities it will provide and to identify gaps or barriers that may need to be addressed. The general standards for training programs required by external accrediting bodies must be rigorously maintained to ensure trainees can meet all required learning outcomes. In addition, attention must be paid to the quality of the experience and relationships with the local community, as these greatly influence whether a trainee eventually chooses to settle and practice within that community [8,19].

Because physicians and other professionals at distributed sites are essential to implementing DME, academic departments must be respectful and attentive as they engage and integrate distributed faculty into their teaching and scholarly activities [3,20-22]. Careful consideration of community engagement is key when developing DME programming, given the community preceptors’ pivotal role in the development, delivery, and ongoing success of DME programming [20,23-27]. Navigating relationships between partners and establishing sound governance structures are essential components of successful DME [3]. Collaborative efforts must incorporate community values, attitudes, and beliefs [28], and all parties should be sensitive to potential challenges that can arise in communications between physicians practicing within the primary academic center and those practicing outside of it [29].

### Organizational Context

Dalhousie University’s Faculty of Medicine has 2 campuses. Dalhousie Medical School based in Halifax, Nova Scotia, Canada opened approximately 150 years ago in 1868. A regional campus Dalhousie Medicine New Brunswick was established in 2010 in Saint John, New Brunswick, Canada. A third distributed campus is planned and will begin accepting students in Cape Breton, Nova Scotia by 2025. Dalhousie University requires anyone completing evaluations of medical trainees to have a faculty appointment, which may be either a continuing appointment or a limited-term appointment.

Clinical training in psychiatry at Dalhousie University comprises both UGME and PGME. In Nova Scotia, there are 2 health authorities where learners may be placed for clinical rotations: IWK Health and Nova Scotia Health. IWK Health provides secondary care to children, youths, and women regionally, tertiary care provincially, and quaternary care for the Maritimes. Nova Scotia Health is divided into 4 geographic zones serving the province: eastern, western, northern, and central. The central zone includes Halifax Regional Municipality, the capital of the province and the largest urban center with a population of 440,000 in 2021 [30]. Dalhousie University’s main campus is in Halifax, and this is where the main teaching hospitals are situated. The central zone also contains the majority of the province’s psychiatrists. The DME located in New Brunswick provides clinical education through the Horizon Health Network. Preclinical medical education is based in Saint John, and 4 of the province’s 7 administrative health zones participate in the clinical education of Dalhousie University’s medical students: zones 1 (Moncton), 2 (Saint John), 3 (Fredericton and River Valley area), and 7 (Miramichi).

In both provinces, psychiatrists at distributed sites make valuable contributions to the clinical education of medical students and residents, both psychiatry and nonpsychiatry. Eventually, the department also plans to extend its recently launched international fellowship training program to DME sites in the 2 provinces.

In contrast, psychiatry residency training at Dalhousie University has been more centralized with the majority of time spent in Halifax. While Dalhousie University’s psychiatry residents rotate for short periods of time through Saint John, New Brunswick, and Kentville, Nova Scotia, the residency program does not currently meet the rural training requirements as set out by the PGME office, which states 10% of postgraduate training should occur in DME learning sites. To meet this requirement, Dalhousie University’s psychiatry residency program has recently expanded its training outside of Halifax to include an inpatient rotation at the Cape Breton Regional Hospital in the eastern zone in their second year of training, a geriatric experience in outpatient clinics in western zone in their third year of training, and a 3-month elective requirement in the Maritimes in their final year of training. Geriatric and child and adolescent psychiatry residency (years 5 and 6) have mandatory DME experiences in rural zones. Nonetheless, the proportion of training outside Halifax is still lower than is desirable.

Greater involvement of distributed sites also offers an opportunity for increasing the training capacity of the residency program. Recently, the Government of Nova Scotia has asked Dalhousie University’s Faculty of Medicine to increase the overall number of psychiatry residency training spots in order to address existing vacancies and meet the projected needs of the growing population of Nova Scotia. Similarly, the New Brunswick government is also interested in developing a New Brunswick–based residency program to assist with recruitment and retention of psychiatrists.

These factors (educational needs, institutional requirements, and political priorities) align with the principles underpinning the Dalhousie University Department of Psychiatry’s Transformational Plan, which was introduced in 2021. Through this plan, the department seeks to extend its academic mandate to enhance medical education, research, and knowledge translation activities at distributed learning sites in Nova Scotia and New Brunswick.

### Environmental Scanning

This paper describes a protocol for an environmental scan of DME sites in Nova Scotia and New Brunswick where the Department of Psychiatry’s faculty members are situated. With this environmental scan, we seek to understand the existing resources and determine what is required to fully integrate DME sites into the academic mandate of the Department of Psychiatry.

Environmental scanning has been used as an effective method of gathering information for a variety of purposes. It is defined as “the acquisition and use of information about events, trends, and relationships in an organization's external environment, the knowledge of which would assist management in planning the organization's future course of action” [31]. Gathering, analyzing, and applying information garnered from environmental scans are essential for strategic planning purposes and organizational effectiveness, as it assists with identifying, assessing, and responding to emerging risks and opportunities [32].

There are process models and conceptual frameworks developed for environmental scanning in the business and educational sectors [33-36]; however, the literature lacks methodological direction on how to go about designing and implementing this strategy to guide research and practice in DME. Environmental scanning is crucial in informing strategic planning and decision-making within organizations, and to the best of our knowledge, no framework has been developed to guide this methodological approach in the DME context within psychiatry training. To this end, we have developed a comprehensive environmental scan protocol built upon previous methodologies [31,37].

## Methods

### Study Design

This study is an environmental scan that adopts a formal information search approach [31] and an explanatory design [37], including quantitative and qualitative data. Formal information search (ie, when a deliberate or planned effort is made to obtain specific information) is often used as an environmental scanning mode [31,33]. The information search is structured according to a pre-established methodology and is focused on systematically finding and retrieving detailed information, usually from sources that ensure data quality and accuracy, in order to provide a basis for developing a decision or course of action [31]. The formal information search approach consists of several interrelated processes that determine the relevance of information, how it will be collected, organized, and stored, what products or services will be developed, and how it will be disseminated and used [38].

Due to the lack of standardized recommendations regarding the methodology to be used in environmental scans using a formal information search approach, the explanatory design was added. The overall purpose of this design is to collect qualitative data to help explain or build upon initial quantitative results [37]. This design starts with the collection and analysis of quantitative data followed by a subsequent collection and analysis of qualitative data.

### Study Settings and Participants

This study will include participants from the 8 administrative health zones (4 in Nova Scotia and 4 in New Brunswick) currently involved in medical education. Dalhousie University Faculty of Medicine DME sites comprise mental health and addiction treatment facilities in the eastern, western, and northern zones of Nova Scotia and 4 health zones of New Brunswick. All psychiatrists at DME sites will be targeted for inclusion in the quantitative study. Based on data obtained from limited-term faculty appointments within the Dalhousie University Department of Psychiatry, it is estimated that approximately 120 psychiatrists are working at DME sites throughout Nova Scotia and New Brunswick. The exact number of psychiatrists working in the central zone of Nova Scotia is unknown, and not all psychiatrists at DME sites in Nova Scotia and New Brunswick have Dalhousie University faculty appointments. Thus, it is difficult to estimate the exact sample size for all psychiatrists working at DME sites in the 2 provinces. A representative sample of psychiatrists working at DME sites, from each health zone in Nova Scotia and New Brunswick, will be invited to participate in the focus group discussions. Purposive sampling will be used to identify subjects for key informant interviews. The distributed sites range in size from small rural communities to medium-sized regional centers, and representing the views of faculty in both types of settings will be an important consideration.

### Study Procedures and Data Collection

#### Overview

**Figure 1:**
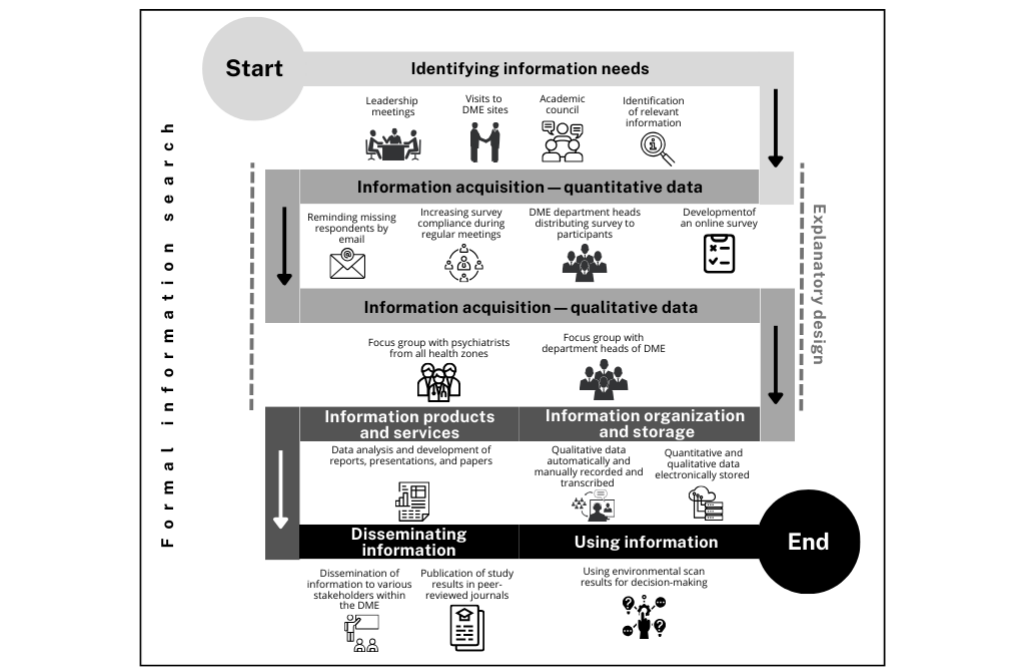
Overview of the proposed environmental scan framework. DME: distributed medical education.

The procedures of the environmental scan framework will be organized according to 6 interrelated processes or phases, as proposed by Choo [31] and explained as follows. The explanatory design [37] is integrated into these processes (Figure 1).

#### Identifying Information Needs

In 2022, a series of meetings were held between the leadership of the Department of Psychiatry and the Faculty of Medicine to generate possible options for increasing residency training capacity. Additionally, the head of the department of psychiatry traveled to the distributed learning sites across Nova Scotia and New Brunswick, meeting with the chiefs of the relevant zones. These tours culminated in the establishment of the Nova Scotia and New Brunswick Psychiatry Academic Council, with membership including the zone chiefs for psychiatry at 8 administrative health zones in the 2 provinces, the chief of psychiatry for the IWK Health authority, the directors of education and research, and the chief operating officer for the Department of Psychiatry. Items discussed during the inaugural meeting of the new Psychiatry Academic Council, which was attended by the dean of the Faculty of Medicine at Dalhousie University and the associate dean for Dalhousie Medicine New Brunswick, included the identification and refining of the categories of information that would be needed for this environmental scan. Following this consensus meeting, the categories of information identified as relevant for this environmental scan were demographics, mental health services and scope of practice, medical education, infrastructure, continuing professional development, and research and knowledge translation. These categories are depicted in the Outcome Measures section.

#### Information Acquisition

Information acquisition is a critical step in the environmental scan process. It involves obtaining relevant data from the right sources that can provide insights into the various factors that may impact an organization's operations, success, and sustainability. Effective information acquisition is essential for conducting a comprehensive and accurate environmental scan. Without access to reliable and relevant data, the scan may miss important trends and risks that could have significant implications for the organization. By contrast, a thorough and well-executed information acquisition process can help organizations stay ahead of emerging risks, take advantage of new opportunities, and make informed strategic decisions [38].

Following are the steps that will be taken during the information acquisition phase of this environmental scan, in which first quantitative and then qualitative data will be collected.

- Acquisition of quantitative data: Based on the information previously identified, a web-based survey was created, piloted, revised, and adopted. A link to the web-based survey will be sent to the clinical department heads for all the administrative health zones, and they will be asked to distribute the survey link to psychiatrists working within their zones. To maximize the survey response rate, engage community-based faculty, and encourage their participation in further program development, the clinical chiefs will be encouraged to set aside 15 minutes of their monthly zone psychiatrists’ meetings for their psychiatrists to complete the survey. In this way, the survey can be completed as a group activity, with each psychiatrist given time to complete the survey confidentially on their personal cell phone or computer. For those not attending the meeting, a second electronic reminder will be sent out to ensure survey recipients complete the survey if they have not done so already. The web-based survey is powered by OPINIO (Object Planet), a web-based survey tool that provides a framework for authoring, distributing, and reporting surveys, which is licensed by Dalhousie University; by completing and submitting the anonymous web-based survey, participants agree to participate in this study. A brief study information leaflet and informed consent will be displayed on the initial page of the survey. The survey comprises 64 multiple-choice questions divided into 5 sections: demographics, mental health services and scope of practice, medical education, continuing professional development, and research and knowledge translation. A final section about study participation includes one question, about the willingness to participate in the focus group. The time expected for survey completion is 15 minutes.
- Acquisition of qualitative data: All the zone clinical department heads in New Brunswick and Nova Scotia will be invited by email to participate in a focus group. A second focus group will be organized for a representative sample of psychiatrists working in the zones who are not clinical department heads. For this group, an email will be sent to all the psychiatrists working at distributed learning sites (excluding the clinical department heads), inviting them to consider participating in a focus group. If interest exceeds demand, block random sampling will be used to select 2 representatives from each administrative health zone. Each of the 2 focus groups will be held over Zoom (Zoom Video Communications), recorded and transcribed verbatim. A transcriptionist will be hired to ensure the transcriptions recorded are accurate. If data saturation (ie, the point in data collection where interviews are no longer generating new information) and sufficiency (ie, that takes into account both individuality and the fact that information is shaped by social factors) [39] are not achieved following the analysis of the qualitative data collected through focus group sessions, psychiatrists who did not participate in the group sessions will be invited to participate in individual interviews until data saturation is achieved. The sample selection method for both focus group sessions and individual interviews (if needed) will involve using a purposive sample, entailing all psychiatrists to participate in the activities. The focus group guide comprises 12 questions, divided into 2 sets of 6 questions. The first set of questions addresses education, covering barriers and facilitators in establishing a distributed learning site, needs or gaps related to the proposed rotation, opportunities that a psychiatry postgraduate education site in your zone would offer learners and the local community, ways to promote and measure faculty engagement, and indicators or outcome measures for ongoing quality assurance and program improvement. The second section focuses on research and scholarship, including barriers and facilitators for establishing research programs, the connection between research and clinical care, personal benefits of research participation, methods to promote and sustain research and scholarly work at your site, and indicators or outcome measures for ongoing research initiatives. The same guide will be used in the individual interviews if those are needed.

#### Information Organization and Storage

During the environmental scan process, it is important to organize and store information so that it can be easily accessed and analyzed to assist with strategic planning and decision-making. When the information is collected electronically (ie, web-based surveys), the organization and storage of the information are superior because it enables easier and more efficient management of the collected large amounts of data. Electronic data can be easily organized, stored, and retrieved when necessary, saving time and resources. In addition, electronic data are less susceptible to errors and can be easily shared with team members who may require access. The proposed environmental scan collects quantitative data using the OPINIO platform, an electronic survey platform that enables the collection and management of survey data. OPINIO can generate reports and export data in multiple formats automatically, facilitating data analysis and visualization. Qualitative data from the focus groups and key informant interviews will be generated automatically by the Zoom platform and manually by a transcriptionist. The data will be stored on a secure drive at Dalhousie University, ensuring its safety and confidentiality. Data stored in Dalhousie University's OPINIO server and the university's secure drive are only accessible to authorized users, thereby protecting sensitive data from theft or loss.

#### Developing Information Products or Services

Developing information products or services entails transforming the data collected during the information acquisition and organization phases into information that can inform strategic planning and decision-making. This step involves analyzing and synthesizing the data collected during the preceding phases into products or services that the organization's decision makers can use. Examples of information products or services that may be developed during this step include reports, presentations, dashboards, and other digital or paper-based visualizations that provide clear and concise information from the environmental scan. This may also involve tailoring the information to the needs of different stakeholders (eg, department heads may require high-level summaries of the key findings, whereas program managers may require more detailed information to support program planning and implementation) [40]. This environmental scan's data analysis strategy will use descriptive and inferential statistics for quantitative data and thematic analysis for qualitative data. The analysis methodology is described in greater detail in the Data Analysis Plan section of this protocol. Following data analysis, PowerPoint (Microsoft Corp) presentations, technical reports, and papers will be prepared by the research team.

#### Disseminating Information

In the information products and services phase, the aim is to share the developed products or services with the environmental scan participants and other stakeholders to aid strategic planning and decision-making. By spreading information, organizations can raise awareness of critical findings and gain support for necessary actions. Communication channels such as reports, presentations, meetings, newsletters, and intranet sites can be used for internal dissemination. The key findings may also be relevant to external stakeholders, including funders, partners, and regulatory bodies, and sharing them can establish relationships and position the organization as a leader. It is crucial to evaluate the effectiveness of the dissemination procedure to ensure that the key findings have been communicated successfully. Such evaluations can identify areas of improvement for future environmental scans [31-33,38]. Findings from this study will therefore be disseminated to the academic psychiatric and medical community and health and medical education policy makers through technical reports to governments of Nova Scotia and New Brunswick, peer-reviewed publications, and regional, national, and international conference presentations.

#### Using Information

The use of information is the final step in the environmental scanning process. It entails several analytical and decision-making processes, such as identifying patterns and relationships in data, assessing the implications of different scenarios, prioritizing opportunities and challenges, and developing action plans to address them. To ensure that the environmental scan insights are considered from multiple perspectives, involving a diverse range of stakeholders in using the information is critical. It is also vital to regularly monitor and update the information to ensure the organization is aware of changing trends and emerging risks [41]. Findings from this environmental scan will be used in the second development phase of the expansion of DME that will involve the distributed sites in planning and preparing for potential separate-entry distributed psychiatry residency programs as well as expansion of the Canadian and International Fellowship programs to distributed learning sites in line with the Transformational Plan for the Department of Psychiatry.

### Outcome Measures

The environmental scan outcome measures were identified in the initial phase of the environmental scan (ie, identifying information needs). They fall into 6 categories:

- Demographics: Participants will provide their demographic information, including their province and health zone of work, type of medical graduation, type of specialist training, academic appointment within the Department of Psychiatry at Dalhousie University, current academic rank, and gender identity.
- Mental health services and scope of practice: Information about participants’ mental health service work, primary and secondary specialization or scope of practice, the practice of psychotherapy, and primary and secondary modes of payment for psychiatric services delivered.
- Medical education: This set of data comprises information about participants’ formal medical education training, level of medical education qualification they have received, their knowledge and skills in medical education, areas of medical education they would like to participate in, clinical training and supervision of learners they currently participate in, years of experience providing clinical training or supervision to medical learners, familiarity with the Royal College of Physicians and Surgeons of Canada Competency by Design for residency training, and availability to participate in formal training to facilitate involvement in psychiatry residency training.
- Infrastructure: Opinions on the infrastructure and team set-up for psychiatry resident training, as well as the willingness to participate in clinical training and supervision, offer lectures or skill-based teaching to psychiatry residents and evaluate their skills through skill-based examinations. The barriers that hindered the respondents from contributing to the training and supervision of psychiatry residents, as well as the potential benefits of having psychiatry residents train on their service, will also be assessed.
- Continuing professional development: The frequency of attendance to the Department of Psychiatry weekly academic grand rounds and the participants' interest in attending and presenting in the grand rounds will be measured. Participants will also be asked about past and future participation in professional development programs organized by the Department of Psychiatry.
- Research and knowledge translation: The survey will assess the respondents' formal research training, level of research qualification received, interest in exploring opportunities for formal research training, and participation in current and preferred mental health or clinical translational research. The survey also assessed the barriers the participants perceived would hinder psychiatrists from participating in research and knowledge translation activities.

### Data Analysis Plan

Data analysis will take place immediately after the phase of information organization and before the development of information products and services.

Quantitative data will be analyzed with SPSS (version 28; IBM Corp) using descriptive and inferential statistics. Demographic data and responses to selected questions will be summarized and presented as numbers and percentages. Depending on the outcome of the descriptive analysis, the chi-square test will be used to assess differences in responses between participants based on their province of work or administrative health zones. Binary or multinomial logistic regression analysis may be used to identify key barriers and enablers for expanding UGME and PGME, continuing medical education, research and knowledge translation at distributed learning sites.

Qualitative data will be analyzed thematically using the NVivo software (version 12; QSR International). Thematic analysis, commonly used in qualitative research, draws upon grounded theory [42] and content analysis [43]. Thematic analysis will be used for identifying and reporting on the major ideas expressed. Initially, transcribed data will be independently coded for themes by 2 investigators. These codes will be reviewed and revised jointly by the 2 investigators through a consensus process and then reapplied to the documents. Themes and subthemes will be created for identified barriers and facilitators for the expansion of UGME and PGME, continuing medical education, research and knowledge translation at distributed learning sites. Verbatim quotes from participants for each of the themes will be provided.

### Ethical Considerations

This protocol has been submitted to the Dalhousie University Research Ethics Board and received an exemption from Research Ethics Board oversight according to the Tri-Council Policy Statement Ethical Conduct for Research Involving Humans article 2.5 [41]. Prior to completing the web-based surveys, all participants provided explicit consent. The informed consent presented on the initial page of the web-based survey included an explanation of the voluntary and anonymous nature of participants' participation in the study with a particular emphasis on the use of their data for research purposes covering primary and secondary data analysis.

## Results

At the time of paper submission, it was anticipated that the findings from this environmental scan would be available within 6 months from the start of data collection. Fortunately, this timeline has been successfully achieved since data collection took place in February and March 2023, and the data have been thoroughly analyzed. One paper, focusing on results related to the psychiatrists' willingness to participate in scholarly activities, was submitted to a peer-reviewed journal in September 2023. Another paper, which delves into the psychiatrists' willingness to engage in academic research activities, is currently under review by the authors and is scheduled for submission to a peer-reviewed journal by the end of this year, with an anticipated publication date in the upcoming year. Study findings will be used to inform the development of policy options and decisions related to the expansion of the psychiatry residency and fellowship training opportunities at DME sites in Nova Scotia and New Brunswick. Options developed and decisions made during the second development phase will be based on information gathered during this initial or information-seeking phase and stakeholder agreements. With this project, we seek to develop an in-depth understanding of existing needs and factors that must be addressed to ensure the success of the proposed expansion of Dalhousie University’s psychiatry residency and fellowship programs using the infrastructure and human resources at distributed learning sites. By identifying challenges and opportunities in advance of the expansion of psychiatry training into a rural area and defining outcome measures to be tracked over time, it will be possible to design and implement processes and structures that will enhance the educational experience of residents and fellows, encourage faculty engagement, and facilitate ongoing program improvement.

## Discussion

This paper describes a protocol for conducting an environmental scan at Dalhousie University's DME sites within the Departments of Psychiatry in Nova Scotia and New Brunswick. It seeks to assess existing resources and determine the necessary steps for the expansion and incorporation of DME sites within the academic framework of the Department of Psychiatry. The chosen method was environmental scanning, well-known for its efficiency in gathering information. Nevertheless, none of the existing frameworks have been designed specifically to guide DME research in psychiatry. As a result, this paper presents a comprehensive environmental scan protocol developed by adapting existing methodologies, combining a formal information search strategy with an explanatory design that includes quantitative and qualitative data.

The protocol holds several strengths that considerably enhance its efficacy and potential impact. Its comprehensive approach facilitates the collection of key factors relating to psychiatrists' willingness to engage in research and scholarly activities, which are essential for the expansion of DME. By incorporating diverse viewpoints and experiences, the protocol comprehensively represents the target population. In addition, its consistency with the existing literature provides a firm foundation for interpreting findings and making meaningful comparisons. In addition, the protocol's innovative approach effectively resolves information gaps in the DME field, providing a unique opportunity to discover insights and trends that may influence the future of psychiatric education.

However, it is essential to acknowledge certain limitations of this protocol. Sample selection methods are dependent on the participation and availability of psychiatrists. While efforts will be made to invite all eligible psychiatrists, logistical obstacles or scheduling conflicts may limit the sample size. Another limitation is the use of self-reporting surveys and focus group sessions for data collection. Participants may provide socially desirable responses or incorrect information.

In addition, the collected data are contingent on the participants' perceptions, which may not accurately reflect the situation. Despite the protocol's comprehensive data collection and analysis approach, it does not account for unanticipated variables such as health care policy or socioeconomic conditions that may influence the results.

Insights gained from this project will be used to enhance the quality and delivery of DME in Nova Scotia and New Brunswick. In addition, the information gathered will help answer important and timely questions regarding how training programs can engage psychiatrists outside the academic center and optimize semiurban and rural training experiences. This may be valuable information for other psychiatry and medical departments wishing to expand their residency and fellowship programming beyond tertiary care centers.

## Abbreviations

DME: distributed medical education
PGME: postgraduate medical education
UGME: undergraduate medical education

## Data Availability

The data sets generated during or analyzed during this study are available from the corresponding author upon reasonable request.
